# TFAP4 Promotes Hepatocellular Carcinoma Invasion and Metastasis via Activating the PI3K/AKT Signaling Pathway

**DOI:** 10.1155/2019/7129214

**Published:** 2019-06-10

**Authors:** Tao Huang, Qi-Feng Chen, Bo-Yang Chang, Lu-Jun Shen, Wang Li, Pei-Hong Wu, Wei-Jun Fan

**Affiliations:** ^1^Department of Minimally Invasive Intervention, State Key Laboratory of Oncology in South China, Collaborative Innovation Center for Cancer Medicine, Sun Yat-sen University Cancer Center, Guangzhou 510060, China; ^2^Department of Vascular Interventional Radiology, The Third Affiliated Hospital of Sun Yat-sen University, Guangzhou 510630, China

## Abstract

Transcription factor activating enhancer binding protein 4 (TFAP4) is established as a regulator of human cancer genesis and progression. Overexpression of TFAP4 indicates poor prognosis in various malignancies. The current study was performed to quantify TFAP4 expression as well as to further determine its potential prognostic value and functional role in patients with hepatocellular carcinoma (HCC). We identified that the expression of TFAP4 mRNA in 369 tumor tissues was higher than that in 160 normal liver tissues. Upregulated TFAP4 expressions were discovered in HCC cell lines compared to the healthy liver cell line, and similarly, the levels of TFAP4 were higher in tumor tissues than its expression in paratumor tissues. High mRNA and protein expression of TFAP4 was associated with worse overall survival (OS) and disease-free survival (DFS). Additionally, TFAP4 expression emerged as a risk factor independently affecting both OS and DFS of HCC patients. Functional studies demonstrated that TFAP4 increased HCC cell migration and invasion. Further investigations found that TFAP4 promotes invasion and metastasis by inducing epithelial-mesenchymal transition (EMT) and regulating MMP-9 expression via activating the PI3K/AKT signaling pathway in HCC. In conclusion, our study demonstrated that TFAP4 is a valuable prognostic biomarker in determining the likelihood of tumor metastasis and recurrence, as well as the long-term survival rates of HCC patients. Exploring the regulatory mechanism of TFAP4 will also contribute to the development of new prevention and treatment strategies for HCC.

## 1. Introduction

Hepatocellular carcinoma (HCC) is the most important type of liver cancer [1] and is responsible for being the 2^nd^ most frequently encountered cause of global cancer-associated mortalities [2]. Surgical resection of the affected liver lobes is an effective treatment modality for early stage HCC, and over the years, prognosis has improved considerably with the continued improvement of operative skills and instruments [3]. Despite this, patients with HCC who have undergone surgical resection do not possess favorable long-term survival rates due to the risks of metastasis and recurrence. Although several tumor biomarkers have been investigated as prognostic modalities in candidates for surgical resection, predictive abilities of these markers are not optimal [4–8]. Therefore, it is essential to identify novel tumor biomarkers, which may provide suggestions for the auxiliary diagnosis and predict clinical outcome. On this basis, further study of its molecular mechanism of function may provide new ideas for the treatment of HCC.

The basic helix-loop-helix leucine-zipper (bHLH-LZ) family is known to participate in regulating cell proliferation and differentiation [9]. The transcription factor activating enhancer binding protein 4 (TFAP4) is a member of this family and has been found to be widely involved in the proliferation, differentiation, metastasis, angiogenesis, and other biological regulatory functions of malignancies [9–11]. Recently, TFAP4 overexpression was reported to confer worse prognosis in various malignancies, such as gastric cancer [12], colorectal cancer [13], prostate cancer [14], and non-small-cell lung carcinoma [15]. Jackstadt et al. [16] also found that TFAP4 can enhance migration and metastasis of tumor cells by activating epithelial-mesenchymal transition (EMT). However, the studies on TFAP4 have rarely been reported in HCC. Although Song et al. [17] found that TFAP4 can enhance tumor-forming ability by activating the Wnt/*β*-catenin pathway in HCC, the effect of TFAP4 on the invasion and metastasis of HCC remains to be studied.

In the present study, we sought to determine TFAP4 expression in HCC cell lines and tissues and performed further analyses to determine its potential correlations with clinicopathological characteristics as well as its ability to prognosticate HCC patients receiving surgical resection. Furthermore, we preliminarily investigated the effect of TFAP4 on HCC invasion and metastasis as well as its possible mechanism. We found that TFAP4 can function as a useful prognostic biomarker and it may promote invasion and metastasis via activating the PI3K/AKT signaling pathway in HCC.

## 2. Material and Methods

### 2.1. Gene Expression Profiling Interactive Analysis

The website of gene expression profiling interaction analysis (GEPIA, http://gepia.cancer-pku.cn/) was used to analyze the TFAP4 mRNA expression in 369 HCC tissues and 160 normal liver tissues derived from the TCGA database and the GTEx database. 362 HCC patients derived from the TCGA database were used to investigate the relationship between prognosis and mRNA expression levels of TFAP4. During the analysis, patients were divided into two groups (high TFAP4 and low TFAP4) according to the median expression of TFAP4. Overall survival and disease-free survival of the two groups were compared. In addition, HCC patients derived from the TCGA database were used to analyze the correlations between the expression of TFAP4 and the expression of EMT markers (N-cadherin, Vimentin), related transcription factors (Snail, Slug, ZEB1, and ZEB2), and MMP-9.

### 2.2. Study Cohort and Follow-Up

217 patients with HCC who received surgical tumor resection without chemotherapy in two medical centers (Guangzhou, China) were enrolled. 101 patients performed the operation in the First Affiliated Hospital of Sun Yat-sen University, and 116 patients performed the operation in the Sun Yat-sen University Cancer Center from 2007 to 2014. In addition, four different HCC cell lines and one healthy liver cell line as well as 10 fresh HCC tumor samples paired with their paratumor tissues were subjected to western blotting and qRT-PCR to determine their respective TFAP4 expression levels. All the patients were diagnosed with HCC based on the World Health Organization criteria. Tumor stage was determined with the tumor-node-metastasis (TNM) as well as the Barcelona Clinic Liver Cancer (BCLC) classification. The Edmondson-Steiner grading system was used to classify the histologic grade of tumor. All patients provided written informed consent upon enrollment into this study which was preapproved by both medical institutions' Ethics Committee.

All patients received once-monthly follow-up for the first six months after the operation and once every three months after. The endpoint of this study was December 31, 2014. Tumor recurrence and metastasis were confirmed by computed tomography (CT) or magnetic resonance imaging (MRI). Overall survival (OS) was determined to be the duration between surgical resection and death. Disease-free survival (DFS) represented the duration between surgical resection to recurrence or metastasis.

### 2.3. Immunohistochemistry

In brief, 4 *μ*m thick tissue sections derived from 217 adult patients were immersed in xylene to remove paraffin before being rehydrated in different ethanol gradations. Following this, sections were autoclaved for 5 min in 10 mM ethylenediaminetetraacetic acid (EDTA, pH 8.0) in order to retrieve antigens. Endogenous peroxidase activity was blocked by immersing sections for 20 min in 3% hydrogen peroxide at room temperature. Next, sections were subjected to overnight incubation with TFAP4 antibody (1 : 50, ab28512, Abcam, Cambridge, UK) at 4°C. The following day, sections were washed and reincubated with a secondary antibody for 30 min at 37°C. Final staining was done with hematoxylin, and sections were then dehydrated and mounted.

### 2.4. Evaluation of Immunostaining

Scoring for staining intensity in each case was based on the following scale: 0, negative; 1, weakly positive; 2, moderately positive; and 3, strongly positive. The proportion of positively stained cells was scored as follows: 1, 0-25%; 2, 25-50%; 3, 50-75%; and 4, 75-100%. The immunoreactive score (IRS) was determined by the sum of the two parameters. Patients were divided into two groups: high (IRS ≥ 4) and low (IRS < 4) TFAP4 expression, with an IRS score of 4 being the median of the total score. All sections were scored independently by two pathologists with more than 10 years of working experience who were blinded to patients' clinical outcome and clinicopathological parameters. Five randomized microscopic fields were used to calculate the average score in each section.

### 2.5. Cell Culture

Human hepatocellular carcinoma (HCC) cell lines (Huh7, MHCC-97H, MHCC-97L, and PLC/PRF/5) and LO2, an immortalized liver cell line, were procured from Cell Bank (Chinese Academy of Sciences, Shanghai, China). Dulbecco's modified Eagle's medium (DMEM, Gibco, USA) was used as a culture medium for cells. All the medium was supplemented with 10% fetal bovine serum. Cells were cultured in an incubator with 5% atmospheric CO_2_ at 37°C.

### 2.6. Quantitative Real-Time PCR

Total RNA was extracted from fresh tissues using the TRIzol agent (Takara Bio, Otsu, Japan). cDNA was synthesized using the PrimeScript RT Reagent Kit (Takara Bio, Otsu, Japan). The real-time quantification PCR was performed using the SYBR Premix Ex Taq Kit (Takara Bio, Otsu, Japan) according to the manufacturers' instructions. The primers used in real-time PCR were as follows: TFAP4 forward primer 5′-GTGCCCACTCAGAAGGTGC-3′ and reverse primer 5′-GGCTACAGAGCCCTCCTATCA-3′; GAPDH forward primer 5′-GGAGCGAGATCCCTCCAAAAT-3′ and reverse primer 5′-GGCTGTTGTCATACTTCTCATGG-3′. The cycling parameters were 95°C for 30 s, followed by 40 cycles of 95°C for 5 s and 60°C for 30 s. Each sample was detected in 3 duplicate wells. GAPDH was used as an internal control. The relative expression of TFAP4 mRNA was calculated using the 2^–ΔΔCt^ method.

### 2.7. Lentivirus-Mediated Plasmid Transfection

The Lenti-TFAP4 expression vector and Lenti-shTFAP4 vector (targeting sequences : 5′-CCTCGGTCATCAACTCTGTTT-3'; control sequences: 5′-TTCTCCGAACGTGTCACGT-3') as well as their respective control vectors were constructed, verified, and purified by Gene Chem (Shanghai, China). MHCC-97L cells were cultured in 24-well plates (about 50-60% confluence) and transfected with Lenti-TFAP4 expression vector and its control vector according to the manufacturer. MHCC-97H cells were transfected with Lenti-shTFAP4 vector and its control vector. Cells were screened and cultured in the medium with 10% FBS and 8 *μ*g/ml polybrene (Sigma, USA). Western blot was used to detect the transfection rate.

### 2.8. HCC Cells Treated with PI3K/AKT Signaling Pathway Inhibitors

MHCC-97L-TFAP4 cells and MHCC-97L-Control cells were cultured in the six-well plates to 50-60% confluence. PI3K/AKT pathway inhibitor LY294002 with a final concentration of 30 *μ*M was added to the MHCC-97L-TFAP4 cells. The other two groups of cells (MHCC-97L-TFAP4 cells, MHCC-97L-Control cells) were added an equal volume of DMSO. All cells were cultured in an incubator with 5% CO_2_ at 37°C. After 48 hours, cells were used for Transwell migration and invasion experiments as well as western blot.

### 2.9. Western Blotting

Cells and fresh tissues were lysed using lysis buffer (Beyotime, Shanghai, China) that contained protease inhibitors after being subjected to washing with cold phosphate-buffered saline (PBS) thrice based on manufacturer's instructions. The BCA method was performed to quantify the protein concentration, with 10% SDS-PGE gel used to separate equal amounts of protein from each other. Subsequently, proteins were transferred onto 0.22 *μ*m polyvinylidene difluoride membranes (PVDF, Millipore, Billerica, MA). Tris-buffered saline (TBS) containing 5% BSA and 0.1% Tween-20 was used to block nonspecific antigens for 90 min at room temperature. After that, membranes were incubated overnight at 4°C with primary antibodies as follows: TFAP4 antibody (1 : 1000, ab28512, Abcam, Cambridge, UK), E-cadherin, N-cadherin, Vimentin (1 : 1000, EMT Antibody Sampler Kit #9782, CST, MA), p-AKT^Ser473^ (1 : 1000, #4060, CST, MA), p-GSK3*β*^Ser9^ (1 : 1000, #5558, CST, MA), GSK-3*β* (1 : 1000, #12456, CST, MA), MMP-9 (1 : 1000, #13667, CST, MA), p-NF-*κ*B^P65^ (1 : 1000, #3033, CST, MA), and GAPDH antibody (1 : 1000, #5174, CST, MA). The next day, membranes underwent three repeated washings with TBST and were incubated again with secondary antibodies (Cell Signaling Technology, MA) at room temperature for 60 min. After 3 final washings with TBST, an enhanced chemiluminescence (ECL) detection system (Merck Millipore, MA, USA) was used to detect protein expressions.

### 2.10. Transwell Invasion and Migration Assays

Transwell invasion assay was performed using 8 *μ*m pore chamber inserts (Corning, USA) coated with Matrigel (BD Biosciences, USA) in 24-well plates. 5 × 10^4^ cells suspended in 300 *μ*l DMEM without FBS were seeded in the upper chamber. The lower chamber was added with 700 *μ*l DMEM that contained 5% FBS. After being incubated at 37°C for 36 hours, the cells that had invaded to the lower surface of the chamber were stained by 4% paraformaldehyde. The invading cells were stained with 0.1% crystal violet and counted under a microscope on five random fields. Similarly, the migration assay was performed in the same way without Matrigel.

### 2.11. Statistical Analysis

Experiments were performed at least three times and representative results were presented. Comparisons of quantitative data were analyzed by the Student t-test between two groups. Data were presented as the mean ± SD. Associations between two gene expressions were analyzed by Spearman's correlation test. The correlations between TFAP4 and clinicopathological characteristics were analyzed with Fisher's exact test and the chi-squared test. The Kaplan-Meier method and the log-rank test were used to analyze the OS and DFS. Univariate and multivariate Cox proportional hazards regressions were carried out to determine factors related to survival. Statistically significant differences were determined when *P* < 0.05.

## 3. Results

### 3.1. Overexpression of TFAP4 in HCC Tissues and Cells

**Figure 1:**
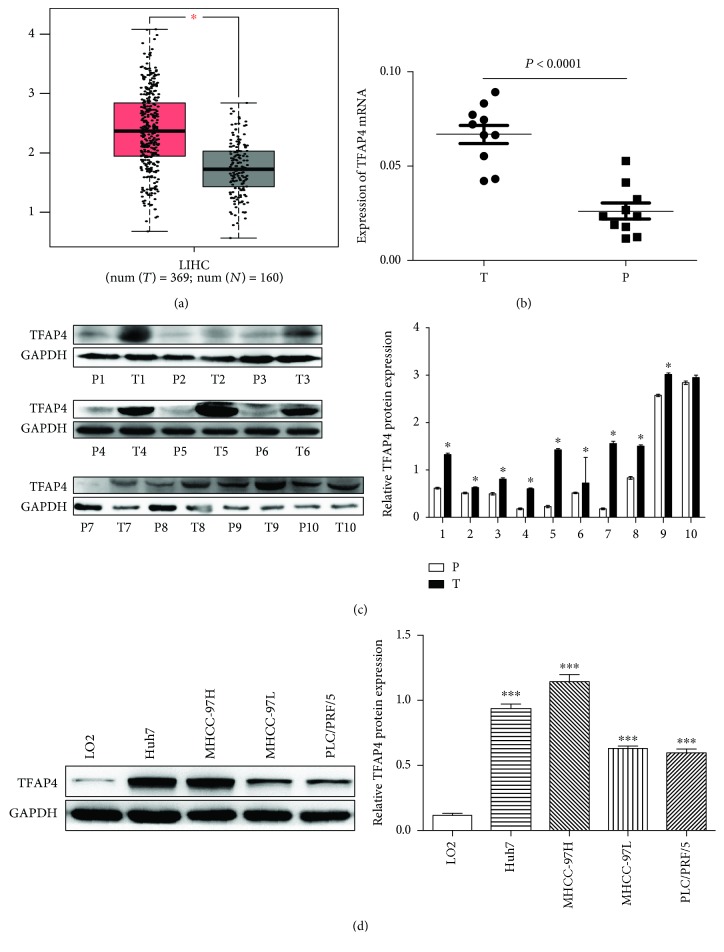
Overexpression of TFAP4 in HCC. (a) GEPIA analyzed the TFAP4 mRNA expression in HCC tissues and normal liver tissues. (b) TFAP4 mRNA expression measured in 10 HCC tumor tissues as well as their adjacent tissues. (c) TFAP4 protein expression measured in 10 HCC tumor tissues as well as their adjacent tissues. (d) TFAP4 protein expression measured in HCC cell lines. HCC: hepatocellular carcinoma; T: tumor tissue; P: paratumor tissue; ^∗^*P* < 0.05; ^∗∗∗^*P* < 0.001.

The website of gene expression profiling interaction analysis (GEPIA) was used to detect the TFAP4 mRNA expression in 369 HCC tissues and 160 normal liver tissues derived from the TCGA database and the GTEx database. The results showed that the expression of TFAP4 mRNA expression in HCC tissues was higher than that in normal liver tissues (Figure 1(a)). Analysis of 10 human fresh HCC samples using qRT-PCR and western blot revealed that tumor tissues possessed higher TFAP4 mRNA and protein expression levels compared to paratumor tissues (Figures 1(b) and 1(c)). Western blot was employed to quantify TFAPA4 protein expressions in four HCC cell lines and a control cell line (LO2). The HCC cell lines showed a higher expression of TFAP4 than the control cell line (Figure 1(d)).

### 3.2. Correlation of TFAP4 Expression with Clinicopathological Parameters and Prognosis in HCC

**Figure 2:**
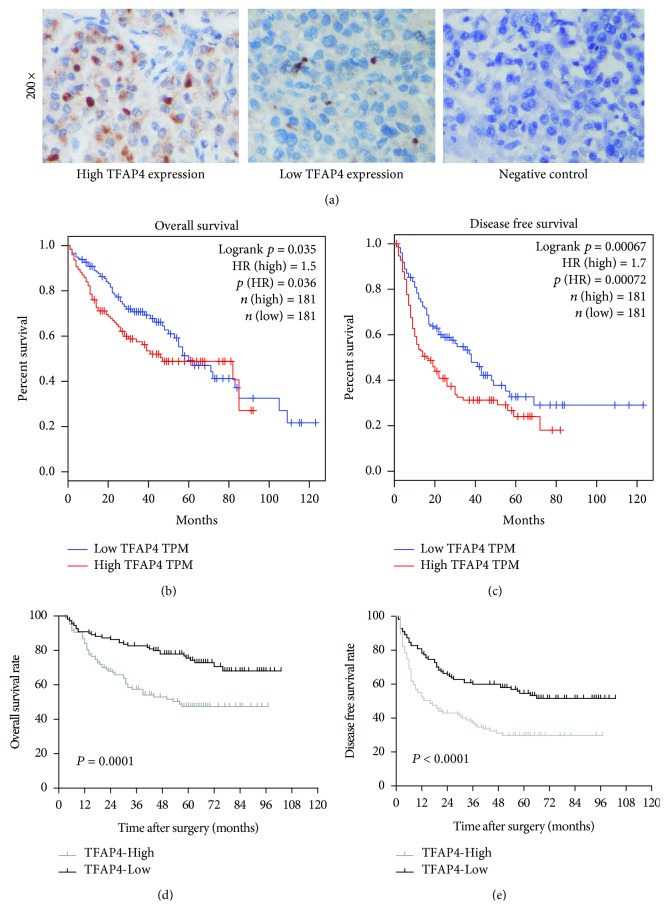
Relationship between TFAP4 expression and prognosis in HCC patients. (a) Immunohistochemical staining in HCC tissues (original magnification ×200). (b) High TFAP4 mRNA expression conferred worse overall survival. (c) High TFAP4 mRNA expression conferred worse disease-free survival. (d) High TFAP4 protein expression conferred worse overall survival. (e) High TFAP4 protein expression conferred worse disease-free survival.

To evaluate the potential ability of TFAP4 to prognosticate HCC patients, 362 patients derived from the TCGA database were subjected to survival analysis. Patients that possessed higher TFAP4 mRNA expressions were found to have worse overall survival rates (*P* = 0.035) and poorer disease-free survival rates (*P* = 0.00067) (Figures 2(b) and 2(c)). To verify these results, TFAP4 expression in 217 HCC tissue samples was further explored with immunohistochemical staining. TFAP4 staining was primarily observed in the nuclei of tumor cells (Figure 2(a)). Overall, TFAP4 expression was found to be positive in 65.0% (*n* = 141) of the 217 HCC patients. 107 cases showed high levels (IRS ≥ 4) of TFAP4 expression, while 110 cases had low levels (IRS < 4) of TFAP4 expression. Patients that possessed higher TFAP4 expressions were found to have worse overall survival rates (*P* = 0.0001) and poorer disease-free survival rates (*P* < 0.0001) (Figures 2(d) and 2(e)).  Table 1 depicts the correlation between clinicopathological parameters and TFAP4 expression.

Univariate Cox regression analysis revealed that vascular invasion, tumor size, number of tumors, BCLC stage, TNM stage, and TFAP4 expression were all strongly correlated with OS rates (Table 2), while gender, vascular invasion, tumor size, number of tumors, AFP, BCLC stage, TNM stage, and TFAP4 expression were strongly correlated with DFS (Table 3). To further determine if TFAP4 was an independent prognostic factor, several potential clinicopathological features with statistical significance (*P* < 0.05) were subjected to further multivariate analysis. Cox proportional hazards regression revealed tumor sizes, TNM stage, and TFAP4 expression to be risk factors that independently determine OS rates (Table 2). Meanwhile, tumor sizes, the presence of vascular invasion, and TFAP4 expression were found to be risk factors that independently affect DFS (Table 3). TFAP4 was found to be an independent prognostic indicator for OS and DFS in HCC patients (HR, 2.712; 95% CI, 1.705-4.314; HR, 2.086; 95% CI, 1.450-3.001, respectively).

HR: hazard ratio; OS: overall survival; CI: confidence interval.

HR: hazard ratio; DFS: disease-free survival; CI: confidence interval.

### 3.3. TFAP4 Promotes the Invasion and Migration of HCC Cells

**Figure 3:**
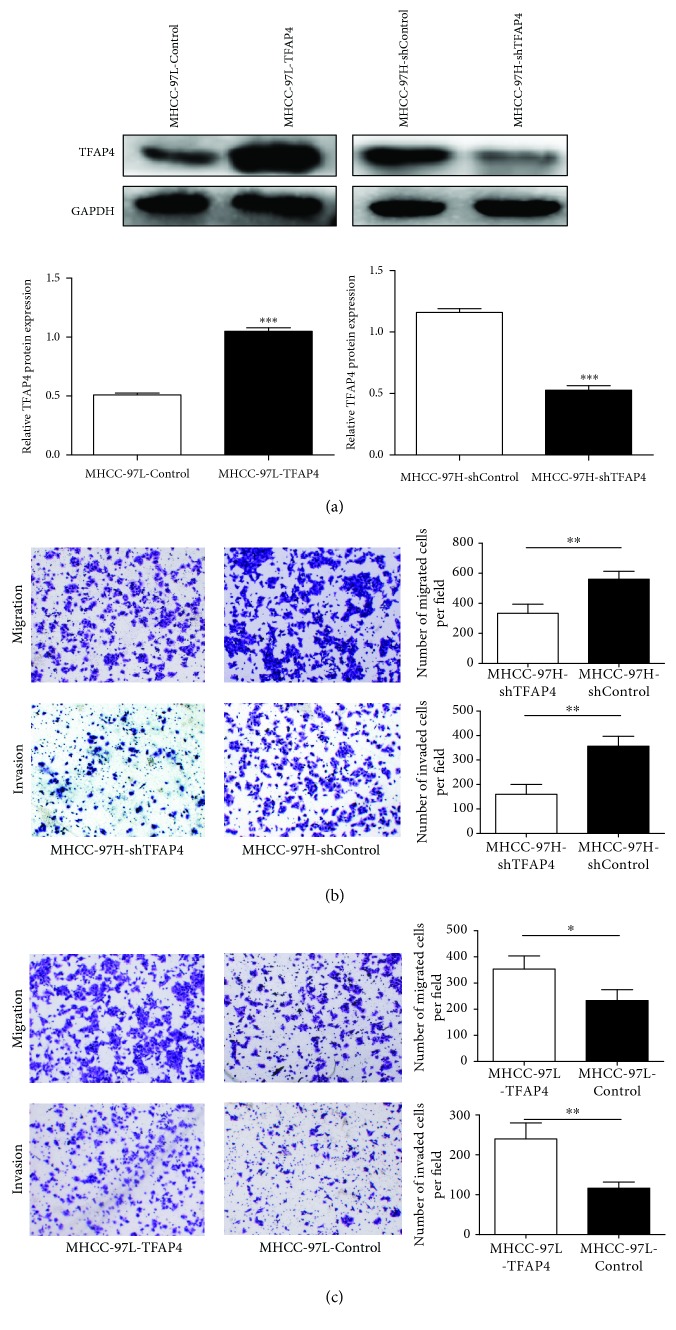
Effects of TFAP4 on HCC cell migration and invasion. (a) Western blot verified the transfection efficiency of TFAP4. (b) Effects of silencing TFAP4 expression on invasion and migration of HCC cells (×100). (c) Effects of overexpression of TFAP4 on invasion and migration of HCC cells (×100). ^∗^*P* < 0.05; ^∗∗^*P* < 0.01; ^∗∗∗^*P* < 0.001.

According to the expression of TFAP4 in HCC cell lines, MHCC-97H cells and MHCC-97L cells were selected to construct stable silencing and overexpressing TFAP4 cell lines, respectively. Western blot results showed that the protein expression level of TFAP4 in the MHCC-97L-TFAP4 cells was significantly higher than that in the MHCC-97L-Control cells. On the contrary, the protein expression level of TFAP4 in the MHCC-97H-shTFAP4 cells was significantly lower than that in the MHCC-97H-shControl cells (Figure 3(a)). Transwell assay demonstrated that the knockdown of TFAP4 repressed cell migration and invasion in MHCC-97H cells (Figure 3(b)). On the contrary, overexpression of TFAP4 increased cell migration and invasion in MHCC-97L cells (Figure 3(c)). These results indicated that TFAP4 could promote the invasion and metastasis of HCC cells.

**Figure 4:**
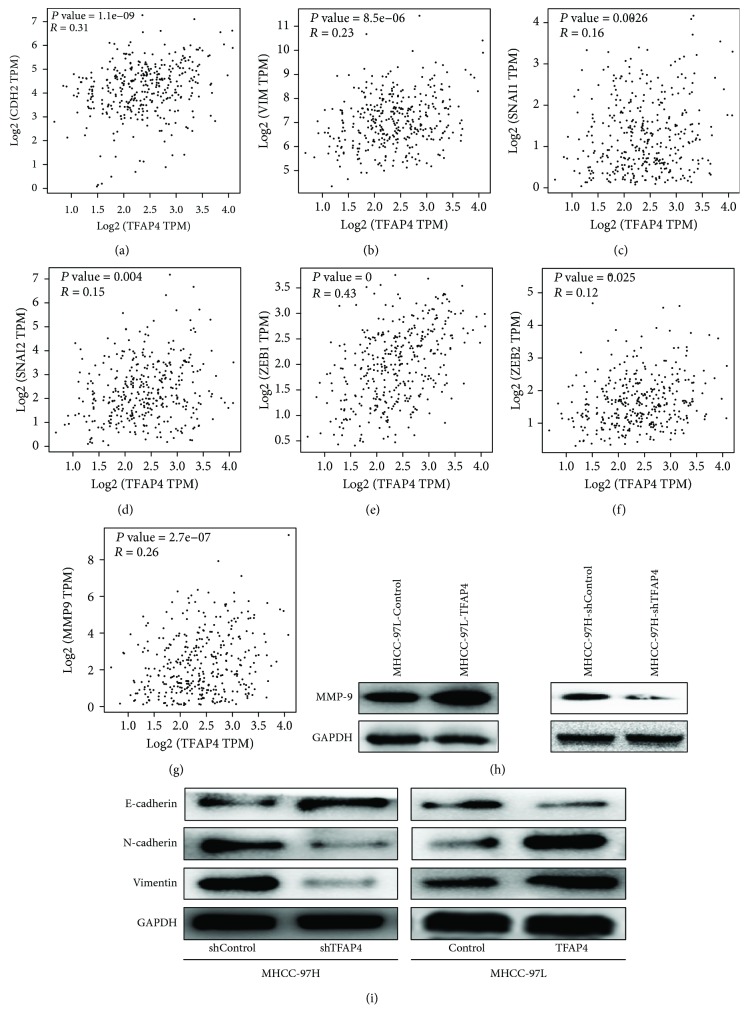
TFAP4 induced EMT and promoted the expression of MMP-9. (a) Correlation between TFAP4 and N-cadherin (CDH2). (b) Correlation between TFAP4 and Vimentin. (c) Correlation between TFAP4 and Snail (Snail1). (d) Correlation between TFAP4 and Slug (Snail2). (e) Correlation between TFAP4 and ZEB1. (f) Correlation between TFAP4 and ZEB2. (g) Correlation between TFAP4 and MMP-9. (h) Protein expression of MMP-9 in HCC cells of each group. (i) Protein expression of EMT markers in HCC cells of each group.

To explore the possible mechanism of TFAP4 promoting invasion and metastasis of HCC cells, we carried out further experiments. The results of GEPIA analysis showed that the expression of TFAP4 was positively correlated with the expression of EMT markers (N-cadherin, Vimentin), related transcription factors (Snail, Slug, ZEB1, and ZEB2), and MMP-9 (Figures 4(a)–4(g)). Western blot results also showed that the expression of the epithelial marker E-cadherin was increased and the expression of mesenchymal markers (N-cadherin, Vimentin) and MMP-9 were decreased in the MHCC-97H-shTFAP4 cells compared with its control cells. On the contrary, the expression of the epithelial marker E-cadherin was repressed and the expression of mesenchymal markers (N-cadherin, Vimentin) and MMP-9 were increased in the MHCC-97L-TFAP4 cells compared with its control cells (Figures 4(h) and 4(i)). These results indicated that TFAP4 could induce EMT and promote the expression of MMP-9.

**Figure 5:**
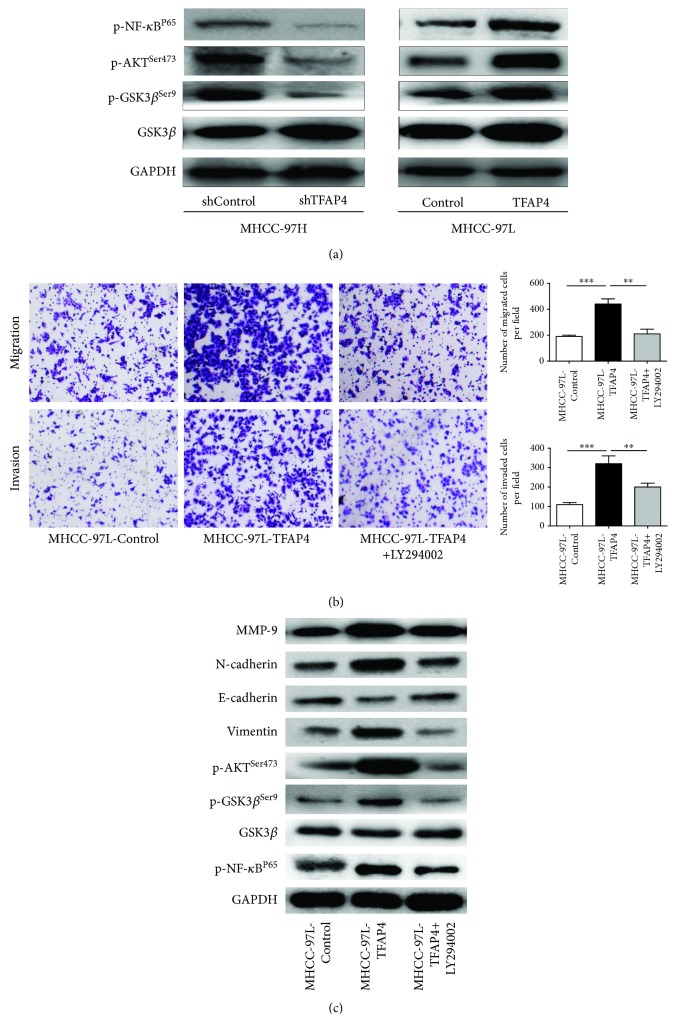
TFAP4 promoted the invasion and metastasis of HCC cells by activating the PI3K/AKT signaling pathway. (a) Western blot detected the protein expression of key components of the PI3K/AKT signaling pathway. (b) Effects of inhibiting the AKT signaling pathway on invasion and migration of HCC cells (×100). (c) Western blot detected the expression of each protein in HCC cells treated with LY294002. ^∗∗^*P* < 0.01; ^∗∗∗^*P* < 0.001.

We then explored the possible mechanism of TFAP4 inducing EMT and promoting MMP-9 expression. Western blot results showed that the expressions of p-AKT^Ser473^, p-GSK3*β*^Ser-9^, and p-NF-*κ*B^P65^ were decreased in the MHCC-97H-shTFAP4 cells compared with its control cells. On the contrary, the expressions of p-AKT^Ser473^, p-GSK3*β*^Ser-9^, and p-NF-*κ*B^P65^ were increased in the MHCC-97L-TFAP4 cells compared with its control cells (Figure 5(a)). These results suggested that TFAP4 could activate the PI3K/AKT signaling pathway. To further verify the results, LY294002 was used to inhibit the PI3K/AK signaling pathway of HCC cells. We found that the abilities of migration and invasion were decreased in MHCC-97L-TFAP4 cells when treated with LY294002 (Figure 5(b)). Western blot results also showed that the expressions of p-AKT^Ser473^, p-GSK3*β*^Ser-9^, and p-NF-*κ*B^P65^ were markedly decreased in MHCC-97L-TFAP4 cells when treated with LY294002, subsequently leading to the increased expression of E-cadherin and the decreased expression of N-cadherin, Vimentin, and MMP-9 (Figure 5(c)). These results further demonstrated that TFAP4 promoted the invasion and metastasis of HCC by activating the PI3K/AKT signaling pathway. Finally, we confirmed that TFAP4 promoted invasion and metastasis by inducing EMT and regulating MMP-9 expression via activating the PI3K/AKT signaling pathway in HCC.

## 4. Discussion

Although there have been considerable advances in methods to diagnose and manage HCC, doctors are still not optimistic regarding the prognosis of this debilitating condition [2]. Early detection, diagnosis, and treatment are key factors that can improve the postoperative survival of HCC patients. Currently, there are few tumor markers for the diagnosis and prediction of recurrence of HCC. AFP is the most commonly used tumor marker of HCC; however, its sensitivity is only 60% [18]. Identifying novel biomarkers may assist in the formulation of novel means to enhance current diagnostic and treatment modalities of HCC. In the present study, we demonstrated that TFAP4 is highly expressed in HCC and may function as a useful prognostic biomarker in HCC patients. We also found that TFAP4 can promote the invasion and metastasis of HCC cells by activating the PI3K/AKT signaling pathway.

TFAP4 has been suggested to encode a c-MYC-inducible repressor of p21 [19], with the inhibition of p21 expression closely associated to a poorer prognosis [20]. Previous studies [12–15] have confirmed that overexpression of TFAP4 predicts poor prognosis in various malignancies, alluding to its potential usefulness as a biomarker that can predict the progression and prognosis of cancer. But in HCC, TFAP4 has rarely been studied. Recently, Song et al. [17] found that TFAP4 was highly expressed in HCC and could promote tumor formation by activating the Wnt/*β*-catenin pathway. However, they only analyzed a small sample size of case data from one medical center and only studied the role of TFAP4 in promoting tumor formation and proliferation of HCC cells. The effect of TFAP4 on the invasion and metastasis of HCC remains to be explored. In this study, we demonstrated an overexpression of TFAP4 in HCC tissues and cells. Furthermore, survival analysis was performed on 362 HCC patients derived from the TCGA database and 217 HCC patients enrolled from two medical centers to determine the relationship between prognosis and TFAP4 expression. Results revealed that higher TFAP4 expression conferred a significantly poorer prognosis, while subsequent Cox regression showed that TFAP4 expression was a risk factor that independently affected both OS and DFS. These results are similar to the previous research, but we also found that the expression of TFAP4 in MHCC-97H cells was higher than that in MHCC-97L cells. These two cell lines were isolated from MHCC-97 cells according to their different metastatic potentials [21]. It indicated that TFAP4 was related to the metastasis of HCC cells. These findings suggested that TFAP4 was an oncogene and functions in the tumorigenesis and metastasis of HCC.

Metastasis of tumor cells is a multistep, complex process, including exit of tumor cells from the primary tumor site, invasion of the extracellular matrix, dissemination via the blood or lymph circulation, and arrival at distant organs. Epithelial-mesenchymal transition (EMT) is a process whereby epithelial cells change into a mesenchymal phenotype, which is an important participant in the process of tumor metastasis [22]. Matrix metalloproteinases (MMPs) are proteinases that can degrade the extracellular matrix. It is generally accepted that tumor invasion and metastasis depend on the role of MMPs [23]. Since patients with high expression of TFAP4 had a higher risk of recurrence and metastasis, we further explored the role of TFAP4 in the invasion and metastasis of HCC. We successfully constructed the stable silenced and overexpressed TFAP4 cell lines. Transwell migration and invasion assay showed that TFAP4 could promote the invasion and metastasis of HCC cells. Wang et al. [24] found that TFAP4 could upregulate LAPTM4B and induce the occurrence of EMT to promote cell proliferation and migration. TFAP4 also promoted metastasis by regulating EMT in colorectal cancer [16]. Through the analysis of the TCGA database, we found that the expression of TFAP4 was positively correlated with the expression of EMT markers, related transcription factors, and MMP-9. Western blot results also confirmed that TFAP4 could promote EMT and the expression of MMP-9. We confirmed that TFAP4 regulated EMT and the expression of MMP-9 to promote the metastasis of HCC cells.

Currently, many signaling pathways have been confirmed to be involved in the regulation of HCC cell invasion and metastasis, the PI3K/AKT signaling pathway is one of the classical pathways. Previous studies have confirmed that activation of AKT signaling pathways could promote EMT in tumor cells [25, 26]. An et al. [27] found that KLF5 can induce EMT via activating PI3K/AKT/Snail signaling in HCC. Jiang et al. [28] confirmed that PRMT9 promoted HCC invasion and metastasis by activating PI3K/Akt/ GSK3*β*/Snail signaling. Our western blot results also showed that TFAP4 could activate the AKT signaling pathway and regulate the activation of GSK3*β* and NF-*κ*B. GSK3*β* is an important component downstream of the AKT signaling pathway, and phosphorylation of GSK3*β* at Ser-9 leads to its reduced activity and promotes EMT [29]. In addition, activation of the AKT pathway leads to the activation of NF-*κ*B in HCC [30]. Previous studies have shown that NF-*κ*B can directly bind to the promoter of MMP-9 and regulate its expression [31–33]. Our experimental results also confirmed that TFAP4 could regulate the activation of GSK3*β* and NF-*κ*B by activating the PI3K/AKT signaling pathway, subsequently leading to the increased expression of MMP-9 and EMT. Furthermore, our western blot results also showed that TFAP4 could not regulate the activation of GSK3*β* and NF-*κ*B after inhibiting the PI3K/AKT signaling pathway with LY294002, nor could it promote the invasion and metastasis of HCC. Taken together, we confirmed that TFAP4 could promote the invasion and metastasis of HCC by inducing EMT and promoting the expression of MMP-9 via activating PI3K/AKT signaling pathways.

In conclusion, we have showed that TFAP4 is a valuable prognostic biomarker in determining the likelihood of tumor metastasis and recurrence, as well as the long-term survival rates of HCC patients. Furthermore, we confirmed that TFAP4 could promote the invasion and metastasis of HCC via activating the PI3K/AKT signaling pathway. Therefore, TFAP4 may serve as a new biomarker of HCC with prognostic value. Exploring the regulatory mechanism of TFAP4 will also contribute to the development of new prevention and treatment strategies for HCC.

## Figures and Tables

**Table 1:** Correlation between clinicopathological parameters and TFAP4 expression in HCC patients.

Clinicopathological parameters	Cases (*n*)	TFAP4 expression		*P* value
		High	Low	
Age (year)				
<60	176	85	91	0.536
≥60	41	22	19	
Gender				
Male	194	100	94	0.056
Female	23	7	16	
HBsAg				
Positive	183	92	91	0.510
Negative	34	15	19	
AFP (*μ*g/l)				
<400	135	65	70	0.661
≥400	82	42	40	
Tumor size (maximal diameter)				
<5cm	107	52	55	0.836
≥5cm	110	55	55	
Number of tumors				
Solitary	181	87	94	0.412
Multiple	36	20	16	
Encapsulation				
Destructed	74	40	34	0.315
Intact	143	67	76	
Vascular invasion				
Yes	191	17	9	0.081
No	26	90	101	
TNM stage				
I-II	184	92	92	0.631
III	33	15	18	
BCLC stage				
0-A	177	84	93	0.251
B	40	23	17	
Differentiated				
I-II	173	88	85	0.363
III-IV	44	19	25	
Micrometastasis				
Yes	16	10	6	0.273
No	201	97	104	

**Table 2:** Univariate and multivariate analysis of the overall survival (OS) in HCC patients.

Variables	Univariate analysis (OS)			Multivariate analysis (OS)		
	*P* value	95% CI of HR	HR	*P* value	95% CI of HR	HR
Age	0.636	0.667-1.941	1.318			
Gender	0.114	0.839-5.128	2.075			
HBsAg	0.517	0.654-2.330	1.234			
AFP	0.193	0.863-2.071	1.337			
Tumor size	<0.001	1.739-4.462	2.786	0.005	1.254-3.528	2.104
Number of tumors	0.001	1.391-3.315	2.148			
Encapsulation	0.455	0.753-1.882	1.191			
Vascular invasion	0.001	1.528-4.732	2.689			
Differentiated	0.066	0.970-2.580	1.582			
Micrometastasis	0.122	0.864-3.454	1.727			
TNM	<0.001	2.187-5.768	3.551	<0.001	1.676-4.955	2.882
BCLC	<0.001	1.466-3.855	2.377			
TFAP4	<0.001	1.500-3.712	2.360	<0.001	1.705-4.314	2.712

**Table 3:** Univariate and multivariate analysis of the disease-free survival (DFS) in HCC patients.

Variables	Univariate analysis (DFS)			Multivariate analysis (DFS)		
	*P* value	95% CI of HR	HR	*P* value	95% CI of HR	HR
Age	0.731	0.584-1.445	0.918			
Gender	0.017	1.182-5.443	2.537			
HBsAg	0.240	0.810-2.317	1.370			
AFP	0.019	1.071-2.184	1.529			
Tumor size	<0.001	1.696-3.543	2.451	<0.001	1.838-3.880	2.671
Number of tumors	0.008	1.142-2.477	1.682			
Encapsulation	0.753	0.729-1.549	1.062			
Vascular invasion	0.001	1.528-4.732	2.689	<0.001	1.696-4.441	2.744
Differentiated	0.106	0.929-2.158	1.416			
Micrometastasis	0.076	0.947-2.989	1.682			
TNM	<0.001	1.732-4.093	2.663			
BCLC	0.014	1.114-2.592	1.700			
TFAP4	<0.001	1.401-2.884	2.010	<0.001	1.450-3.001	2.086

## Data Availability

The data used in this study are available from the corresponding authors upon request.
